# Willingness to pay for haemodialysis among patients with chronic kidney disease in Abuja, Nigeria

**DOI:** 10.1186/s12882-024-03459-4

**Published:** 2024-01-23

**Authors:** Yakubu Adole Agada-Amade, Daniel Chukwuemeka Ogbuabor, Eric Obikeze, Ejemai Eborieme, Obinna Emmanuel Onwujekwe

**Affiliations:** 1 Department of Health Administration and Management, University of Nigeria, Enugu Campus, Enugu, Enugu State Nigeria; 2 National Health Insurance Authority, Abuja, Nigeria; 3 Department of Health Systems and Policy, Sustainable Impact Resource Agency, Enugu, Nigeria; 4 Health Policy Research Group, Department of Pharmacology and Therapeutics, College of Medicine, University of Nigeria, Enugu Campus, Enugu, Nigeria; 5 Department of Psychiatry, Faculty of Medicine, Dalhousie University, Halifax, NS Canada

**Keywords:** Willingness-to-pay, Altruistic willingness-to-pay, Contingent valuation method, Economic evaluation, Haemodialysis, End-stage kidney disease, Nigeria

## Abstract

**Background:**

Evidence of willingness to pay for kidney replacement therapy is scarce in low-middle-income countries, including Nigeria’s Formal Sector Social Health Insurance Programme. The study, therefore, assessed the willingness to pay for haemodialysis among chronic kidney disease patients in Abuja, Nigeria.

**Methods:**

The study adopted a cross-sectional survey design. We used the contingent valuation method to estimate the maximum stated willingness to pay (WTP) for haemodialysis among end-stage kidney disease (ESKD) patients. We obtained informed written consent from respondents before data collection. The socio-demographic characteristics and willingness to pay data were summarized using descriptive statistics. We evaluated the mean differences in respondents’ WTP using Mann-Whitney and Kruskal-Wallis tests. All variables that had *p* < 0.25 in the bivariate analysis were included in the Generalized Linear Model (gamma with link function) to determine the predictors of the WTP for one’s and another’s haemodialysis. The level of significance in the final model was ρ < 0.05.

**Results:**

About 88.3% and 64.8% of ESKD patients were willing to pay for personal and altruistic haemodialysis, correspondingly. The mean annual WTP for haemodialysis for one’s and altruistic haemodialysis was USD25,999.06 and USD 1539.89, respectively. Private hospital patients were likelier to pay for their haemodialysis (β = 0.39, 95%CI: 0.21 to 0.57, *p* < 0.001). Patients attending public-private partnership hospitals were less likely to pay for altruistic haemodialysis than those attending public hospitals (β = -1.65, 95%CI: -2.51 to -0.79, *p* < 0.001).

**Conclusions:**

The willingness to pay for haemodialysis for themselves and others was high. The type of facility ESKD patients attended influenced their willingness to pay for haemodialysis. The findings highlight the need for policies to enhance affordable and equitable access to haemodialysis in Nigeria through pre-payment mechanisms and altruistic financing strategies.

## Introduction

The disease and economic burden of chronic kidney disease (CKD) is high in low- and middle-income countries (LMICs) [1]. The kidney is chronically damaged when its function decreases to a glomerular filtration rate of less than 60 mL/min per 1.73 m2 or the markers of kidney damage, such as albuminuria or haematuria, lasts for at least three months, regardless of the underlying cause [2]. The last stage of CKD, termed end-stage kidney disease, requires kidney replacement therapies (KRTs), such as kidney transplantation or dialysis [1]. Without KRTs, death is inevitable in end-stage kidney disease (ESKD) [1]. About 78% of the 500 million people affected globally by CKD reside in LMICs [3]. The prevalence of CKD in LMICs is 14.3% in the general population and 36.1% in high-risk populations [3]. Africans are likelier to develop CKD and progress to ESKD [4]. The prevalence of CKD was 17.7%, and 6.1% for advanced stages of CKD in sub-Saharan Africa [4]. The prevalence of CKD was 20.4%, with a 300% increase over three decades, while ESKD constitutes about 8–23% of hospital admissions in Nigeria [5].

The economic burden of CKD on patients, providers, government, and society is high. ESKD patients in Nigeria and Burkina Faso face financial hardship from paying for haemodialysis at a cost two or more times higher than the country’s minimum wage [6, 7]. The cost of managing CKD increases substantially with the disease severity [8, 9]. For instance, CKD patients incur a 1.1–1.7-fold and 1.3–4.2-fold increase in per-patient mean annual health care cost transiting within the early and advanced stages of CKD, respectively [9]. In contrast, the health-related quality of life of CKD patients decreases with the advancing stages of CKD [9]. CKD has a high caregiving burden with attendant loss of productivity [10]. Patients with CKD incur non-medical costs such as transportation and loss of productivity from absenteeism, unemployment, and disability [11, 12]. Globally, disability-adjusted life years (DALYs) for CKD rose from 29th in 1990 to 18th in 2019 [13] and would become the 5th most common cause of years of life lost by 2040 [14]. Even though ESKD patients on dialysis only comprise approximately 0.15% of the global population, resources invested in their care constitute 2–4% of national healthcare budget expenditure [8, 15]. Spending on the ESKD population accounts for about 7% of total Medicare expenditures in the USA in 2021 [16]. In China, the economic burden of CKD in 2019 was 1.3% of the Gross Domestic Product (GDP) and 18.8% of total health expenditure [12].

Studies on the willingness to pay for haemodialysis are scarce in LMICs despite escalating healthcare and economic costs related to an increasing burden of CKD. Published studies on the financing of haemodialysis in LMICs indicate that despite the increasing availability of haemodialysis in LMICs, exorbitant costs limit access of ESKD patients to haemodialysis [17]. Most LMICs pay for haemodialysis out-of-pocket [6, 18–20]. Few LMICs publicly fund haemodialysis through the budget or universal health coverage schemes [17]. In Thailand, patients under the universal health coverage scheme can choose either peritoneal dialysis or haemodialysis for long-term kidney replacement therapy with the updated reimbursement policy of the insurance scheme [21]. South Africa adopts a mix of publicly funded haemodialysis using a sliding scale fee strategy and strategic purchasing using health insurance schemes [22]. In Nigeria, most ESKD patients pay out-of-pocket for haemodialysis, given that out-of-pocket spending is the most typical mode of payment for healthcare [1, 6]. Additionally, only 10.8% of Abuja’s population is enrolled in Nigeria’s Formal Sector Social Health Insurance Programme [23]. The National Health Insurance Authority (NHIA) annually covers six haemodialysis sessions [1]. Hence, evidence suggests that full coverage of haemodialysis in benefits of universal coverage schemes can reduce the economic burden of patients with CKD and improve access to CKD care.

Integrating haemodialysis into the benefits package of universal health coverage schemes requires economic evidence, including the willingness to pay (WTP). The WTP measures the value of health benefits of a specific improvement to health by eliciting respondents’ monetary values and preferences [24, 25]. Regarding kidney replacement therapy, the maximum monetary amount the ESKD patients are willing to pay to change their health to an improved state by receiving a dialysis or kidney transplant is a measure of the value of the health benefit derived from the treatment. Evidence of willingness to pay for kidney replacement therapies is scarce, with more studies on kidney transplantation than on haemodialysis [18, 21, 24, 26, 27]. In Thailand, the average WTP for haemodialysis among ESKD patients is one-sixth of the general billing price [27]. Whereas factors influencing WTP for haemodialysis are understudied, price, doctors’ opinion, wealth index, social support, religiosity, ethnicity, employment status, awareness of kidney function, number of years with ESKD, insurance coverage, and patients’ income affected the WTP for a kidney transplant [18, 24, 26, 27].

Therefore, this study assessed ESKD patients’ willingness to pay for haemodialysis and its determinants in Abuja, Nigeria. It also investigated the amounts patients are willing to pay so that people experiencing poverty can receive haemodialysis. The cost of haemodialysis is high and limits access and sustainability in Nigeria. It is imperative to determine how ESKD patients value haemodialysis, which is evidence that could influence improvement in public subsidies for haemodialysis. Hence, this study provides new evidence that can inform a cost-benefit analysis to support the design of appropriate financing policies for chronic kidney disease in Nigeria and other countries intending to reduce the high burden of CKD morbidity and mortality.

## Methods and materials

### Framing of willingness to pay

The study adopted the contingent valuation method, a stated preference method used to assess public preferences by eliciting the WTP values as its main theoretical framework [25]. The WTP approach outlines the maximum amount a person is willing to pay out of their income to reduce the probability of death or gain health improvement [28]. Based on the welfare economic theory, preferences are fixed and exogenous, and consumers act rationally to maximize utility [29]. The welfare economic theory posits that an individual’s maximum WTP measures the benefit to an individual of a service or intervention for the service or intervention [29]. Therefore, individuals opt for treatments only when their WTP for the improvement in their health is equal to or greater than the cost of the treatment [29, 30]. The WTP is related to the individual’s assessment of the intervention’s efficacy or perceived service quality [25]. The WTP technique is suitable for valuing health benefits and setting treatment priorities for non-market goods and services or where regulatory mechanisms or legal constraints limit market choices and where the market price does not accurately reflect the value [24, 29].

Additionally, the study utilized Schwartz’s Norm-Activation Model to explain altruistic WTP, that is, how a person sacrifices his self-interest for the joint benefits of others. Altruism, a motive to enhance other people’s health and well-being [31], is an essential source of non-use value. Altruistic persons are concerned about other people who cannot afford haemodialysis and consider it reasonable to pay some money to protect others. According to Schwartz, the link between personal norms and a specific behaviour is affected by an individual’s awareness of negative consequences and how they ascribe responsibility. Therefore, people with a higher awareness of the need for haemodialysis and responsibility for improving the quality of healthcare services are more likely to pay for its improvement.

### Study area

We studied Abuja, Federal Capital Territory (FCT), Nigeria. The FCT is Nigeria’s administrative and political capital and has the highest social health insurance enrolment. The territory shares boundaries with Niger State to the west, Kaduna State to the north, Nasarawa State to the east, and Kogi State to the south. FCT consists of six Area Councils, consisting of many satellite towns. The population was 2.9 million in 2018, comprising 50.9% males and 49.1% females. Civil service and farming are the predominant occupations. Abuja dialysis centres, accounting for 19% (15) of Nigeria’s 80 functioning dialysis centres, serve not just the FCT but also Nigeria’s entire North-Central region [32]. The study sites were six (6) of the fifteen dialysis centres, including three public, two private and one public-private partnership (PPP) hospitals selected to maximize geographical spread, ownership variation and dialysis coverage.

### Study design

The study adopted a cross-sectional survey design using the contingent valuation method (CVM) with the maximum willingness to pay (WTP) approach.

### Sample size and sampling strategy

The minimum required sample size for this study was 197 using the sample size determination formula for a finite population of ESRD patients receiving haemodialysis in Abuja (*N* = 563), given the proportion of ESRD patients willing to pay for haemodialysis (*p* = 78%) in a previous study [21], 95% confidence limit, allowable error of 0.05 and 10% non-response rate. However, our sample included 230 participants who met the eligibility criteria. The inclusion criteria were consenting adult chronic kidney disease patients with ESKD accessing dialysis care in Abuja. We excluded ESKD patients residing in Abuja but undergoing haemodialysis outside the city, non-consenting ESKD patients and patients receiving haemodialysis for acute kidney injury.

We used a multistage sampling technique to select the participants. The first stage was to select six hospitals from a sampling frame of 15 facilities that offer renal services in Abuja using stratified random sampling: four healthcare facilities in urban/municipal areas and two in satellite towns. The stratification accounted for geographical spread, dialysis coverage, and diverse ownership of hospitals in recruiting facilities. The second stage was the recruitment of patients from the six selected hospitals. We allocated the sample to the six hospitals using probability proportional to size of ESKD patients (private hospital A = 43.9%, private hospital B = 1.3%, public-private partnership hospital = 17.4%, public hospital A = 22.6%, public hospital B = 12.6%, and public hospital C = 2.2%). In each hospital, the eligible patients were selected by simple random sampling.

### Data collection procedure

The data collection took place between July 2019 and February 2020. Data was collected using an interviewer-administered questionnaire. The survey collected data on their socio-demographic characteristics and willingness to pay (WTP) for their haemodialysis and for people experiencing scarcity. The benefit was estimated using the Contingent Valuation Method (CVM) with the maximum Willingness to Pay (WTP) approach. We asked the respondents to state their WTP for haemodialysis. Subsequently, we asked the respondents to state their maximum WTP for a haemodialysis session. The respondents first valued two health states and expressed their WTP for avoiding a decline in health, from a better health state to a worse one with all the disabilities and, ultimately, death. The study used the “bidding game”, which starts with a single bid and increases or decreases following the respondent agreement till the maximum WTP is reached [30]. The bidding process for eliciting WTP for one’s use followed the format:

1. If you will be required to start payment immediately, how much in Naira are you willing to pay for a session of renal dialysis? A = 10,000; B = 15,000; C = 20,000; D = 25,000; E = Nil.
2. What if the price you had to pay for haemodialysis was higher than the amount you stated above? Will you be willing to pay? 1 = yes; 0 = No.
3. What if the cost is lower than you stated above? Will you be willing to pay? 1 = yes; 0 = No.
4. What is the maximum amount you will pay for a haemodialysis session?

For altruistic WTP, we asked respondents to state the maximum amount they were willing to contribute to ensure that people experiencing poverty have access to haemodialysis or comprehensive conservative care.

### Data analysis

Data was analyzed using the SPSS version 20. The study reported the respondents’ socio-demographic characteristics using frequencies and proportions. The proportion of respondents willing to pay any stated amount was reported as a measure of willingness to pay. We converted the costs in Nigerian Naira () to US dollar ($) at the 2018 exchange rate ($1 = 308.5). We cross-tabulated the mean WTP and the respondents’ socio-demographic and health-related characteristics. We evaluated the mean differences in respondents’ WTP using Mann-Whitney and Kruskal-Wallis tests because the Shapiro-Willi test indicated that the monetary data were skewed. All variables that had *p* < 0.25 in the bivariate analysis were included in the Generalized Linear Model (gamma with log link) to determine the predictors of the WTP for one’s and another’s haemodialysis. Generalized Linear Model (gamma with log link) is suitable for analyzing WTP data that violate the assumptions of the linear model fitted using ordinary least squares, including homoskedasticity, normality, and independence. The level of significance in the final model was ρ < 0.05.

### Ethical considerations

The Federal Capital Territory (FCT) Health Research Ethics Committee approved the study’s research protocol (FHREC/2019/01/02/10-01-19). We also obtained administrative approvals from the participating hospitals and informed written consent from respondents at the time of data collection. All data were anonymized before analysis and stored in a secure, password-protected computer.

## Results

### Socio-demographic characteristics of respondents

Table 1 shows the socio-demographic characteristics of the respondents. Most respondents were male, married, resided in the municipality or its satellite towns, employed, and had higher education. Over 50% of patients were at least 60 years old, earned less than USD324.1 monthly income and had one co-morbidity. Just over 40% of patients attended a private hospital.

**Table 1:** Socio-demographic characteristics of respondents (*N* = 230)

Characteristics		Frequency (n)	Percent (%)
Gender	Male	150	65.2
	Female	79	34.3
	Missing	1	0.4
Age (years)	< 40	40	17.4
	40–59	69	30.0
	≥ 60	121	52.6
Marital status	Single	51	22.2
	Married	145	63.0
	Widowed	21	9.1
	Divorced/Separated	8	3.5
	Missing	5	2.2
Residence	Others	24	10.4
	Satellite	90	39.1
	Nearby towns	45	19.6
	Municipality	69	30.0
	Missing	2	0.9
Education	SSCE and below	51	22.2
	Higher education	179	77.8
Employment	No	40	17.4
	Yes	190	82.6
Income	< $324.15	123	53.5
	≥ $324.15	107	46.5
Wealth index	Poor	11	4.8
	Middle	10	4.3
	Rich	209	90.9
Health insurance	No	178	77.4
	Yes	52	22.6
Type of Facility	Private	99	43.0
	PPP	38	16.5
	Public	86	37.4
	Missing	7	3.0
Comorbidity	0–1	135	58.7
	≥ 2	95	41.3
Number of treatment session per month	≤ 3	25	10.9
	4–6	58	25.2
	> 6	141	61.3
	Missing	6	2.6

### Willingness to pay and mean benefits of haemodialysis

**Fig. 1:**
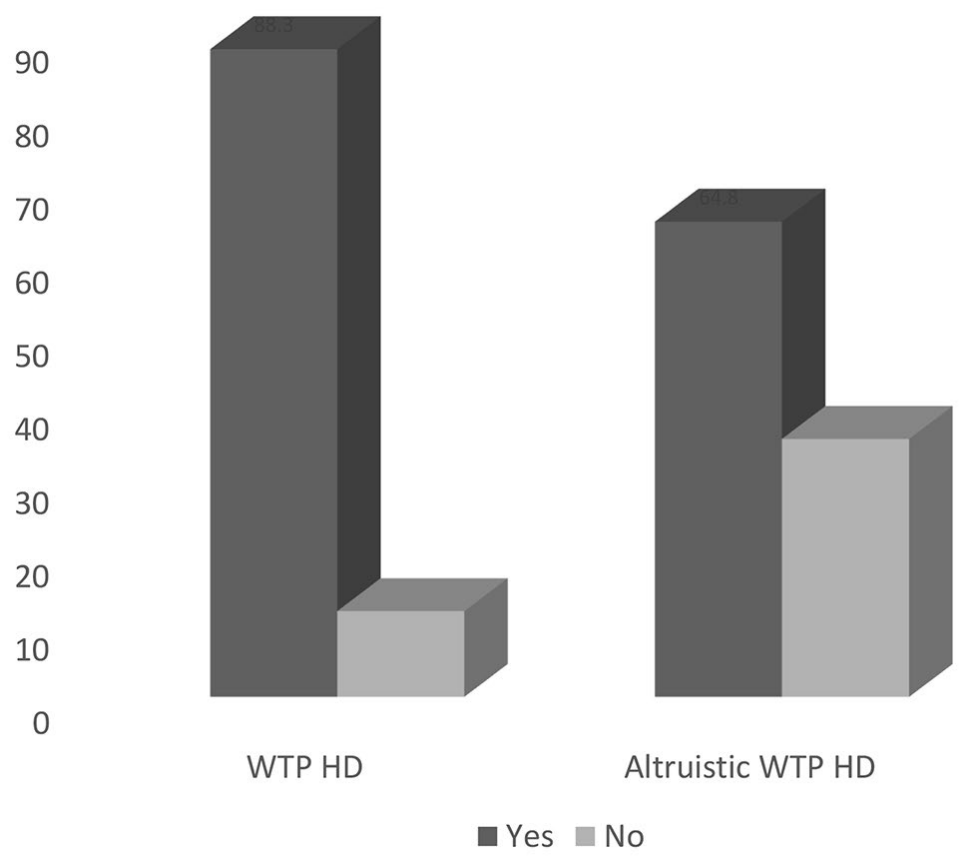
Willingness to pay for haemodialysis for themselves and others among ESKD patients

About 88.3% and 64.8% of patients receiving haemodialysis were willing to pay for their haemodialysis and others, correspondingly (Fig. 1). However, just a quarter of ESRD patients receiving haemodialysis were willing to pay and above the current cost of haemodialysis. Further, 11.7% and 35.2% of ESKD patients were unwilling to pay for haemodialysis for themselves and people experiencing scarcity.

Patients receiving haemodialysis were willing to pay a mean monetary value of USD 25,999.06 for haemodialysis. Regarding altruism, patients were willing to pay a mean monetary value of USD 1539.89.

### Factors associated with willingness to pay for own haemodialysis

Table 2 shows that the mean WTP for own haemodialysis significantly differed by respondents’ type of facility (*p* < 0.001) and number of treatment sessions (*p* = 0.005). Patients receiving haemodialysis in private hospitals had higher WTP than those in public and PPP hospitals. Patients receiving greater than six treatment sessions had significantly higher WTP than those receiving less than six haemodilaysis sessions per month.

**Table 2:** Mean differences in personal and altruistic willingness to pay for haemodialysis

Socio-demographic characteristics		WTP		Altruistic WTP	
		Mean (Std. Dev)	*P*-value	Mean (Std. Dev)	*P*-value
Gender^**+**^	Male	26063.37(15015.39)	0.783	1739.69(3657.89)	0.255
	Female	25383.34(14423.04)		1132.14(1656.17)	
Age (years) ^**++**^	< 40	24886.32(12537.14)		3035.01(7141.76)	
	40–59	22846.45(13857.49)	0.123	1660.49(2150.50)	0.191
	≥ 60	27953.94(15814.11)		1099.27(1661.54)	
Marital status^**++**^	Single	26126.42(12445.11)		2277.04(5928.76)	
	Married	26508.48(15700.84)	0.521	1372.54(1839.14)	0.310
	Widowed	20816.86(863.97)		1650.46(2565.83)	
	Divorced/Separated	23333.18(21832.78)		888.71(1950.17)	
Residence^**++**^	Others	30933.97(16260.09)		3620.05(8487.11)	
	Satellite	26735.82(15033.92)	0.091	1606.68(2333.99)	0.071
	Nearby towns	21825.92(12043.03)		948.26(1419.24)	
	Municipality	25860.41(15606.14)		1207.17(1443.04)	
Education^**+**^	SSCE and below	26614.35(18003.20)	0.568	2121.31(6390.43)	0.058
	Higher education	25853.66(14204.92)		1416.99(1892.28)	
Employment^**+**^	No	27474.88(17029.34)	0.811	2224.91(6385.88)	0.092
	Yes	25677.27(14490.62)		1395.09(1886.47)	
Income^**+**^	< $324.15	26308.77(15573.00)	0.747	1340.23(3957.12)	0.002^*^
	≥ $324.15	25626.18(14235.61)		1742.26(2039.56)	
Wealth^**++**^	Poor	21350.62(19758.11)		1373.31(1570.60)	
	Middle	27179.90(16427.41)	0.331	7153.97(14189.28)	0.998
	Rich	26169.92(14682.01)		1343.73(1886.75)	
Health insurance^**+**^	No	26607.12(15386.31)	0.339	1596.19(3450.08)	0.884
	Yes	23581.61(12930.18)		1316.59(1462.17)	
Type of facility^**++**^	Private	31048.30(16640.41)		2194.86(4362.71)	
	PPP	21823.77(10439.86)	< 0.001^*^	378.14(900.60)	0.001^*^
	Public	19849.66(10864.83)		1690.40(1690.01)	
No of comorbidities^**+**^	≤ 1	26053.33(14699.35)	0.959	1275.39(3684.89)	< 0.001^*^
	≥ 2	25924.15(15324.65)		1900.95(2199.85)	
No of treatment sessions per month^**++**^	≤ 3	25768.23(11678.14)		958.53(1776.71)	
	4–6	19709.19(12578.01)	0.005^*^	979.15(1384.91)	0.066
	> 6	28023.32(15729.70)		1841.38(3672.00)	

^**+**^Mann-Whitney and ^**++**^Kruskal-Wallis tests, ^*^Significance at *p* < 0.05

### Factors associated with altruistic willingness to pay for haemodialysis

Table 2 shows that, significant mean differences in altruistic WTP existed by income (*p* = 0.002), type of health facility (*p* = 0.001), and number of co-morbidities (*p* < 0.001). Patients earning at least USD324 attended private hospitals, and those with two or more co-morbidities were more willing to pay for altruistic haemodialysis. The co-morbidities included diabetes mellitus, hypertension, anaemia, cardiovascular diseases, sexual dysfunction and depression.

### Determinants of willingness to pay for personal and altruistic haemodialysis

Table 3 shows the determinants of WTP for personal and altruistic haemodialysis. Patients receiving haemodialysis in private hospitals were likelier to pay for their haemodialysis (β = 0.39, 95%CI: 0.21 to 0.57, *p* < 0.001). On the other hand, ESKD patients attending public-private partnership hospitals were less likely to pay for altruistic haemodialysis than those attending public hospitals (β = -1.65, 95%CI: -2.51 to -0.79, *p* < 0.001).

**Table 3:** Predictors of personal or altruistic willingness to pay for haemodialysis

WTP category	Parameter		B	Std. Error	95% Confidence Interval		Hypothesis Test	
					Lower	Upper	Wald Chi-Square	Sig.
Personal haemodialysis		(Intercept)	9.90	0.15	9.60	10.21	4121.10	< 0.001
	Age (years)	< 40	0^a^					
		40–59	-0.12	0.11	-0.34	0.10	1.20	0.274
		≥ 60	0.09	0.11	-0.13	0.30	0.63	0.426
	Residence	Others	0.08	0.12	-0.16	0.31	0.41	0.520
		Satellite	0.07	0.09	-0.10	0.24	0.66	0.418
		Nearby towns	-0.02	0.11	-0.24	0.20	0.04	0.839
		Municipality	0^a^					
	Type of facility	Private	0.39	0.09	0.21	0.57	17.80	0.000
		PPP	0.04	0.11	-0.17	0.25	0.16	0.688
		Public	0^a^					
	No of dialysis sessions per month	≤ 3	0^a^					
		4–6	-0.15	0.13	-0.40	0.10	1.34	0.246
		> 6	0.02	0.11	-0.21	0.24	0.02	0.893
		(Scale)	0.23^b^	0.02	0.19	0.28		
Altruistic haemodialysis		(Intercept)	7.98	1.01	6.00	9.97	62.00	< 0.001^*^
	Age (years)	< 40	0^a^					
		40–59	-0.49	0.53	-1.53	0.55	0.86	0.355
		≥ 60	-0.44	0.51	-1.45	0.56	0.75	0.386
	Residence	Others	0.64	0.65	-0.63	1.91	0.97	0.324
		Satellite	0.13	0.34	-0.55	0.80	0.13	0.715
		Nearby towns	-0.16	0.44	-1.02	0.70	0.13	0.716
		Municipality	0^a^					
	Education	SSCE and below	-0.07	0.55	-1.16	1.01	0.02	0.895
		Higher education	0^a^					
	Employment	No	0.34	0.53	-0.70	1.38	0.40	0.525
		Yes	0^a^					
	Income	< $324.15	0.00	0.37	-0.73	0.73	0.00	0.995
		≥ $324.15	0^a^					
	Type of facility	Private	-0.59	0.47	-1.51	0.33	1.56	0.211
		PPP	-1.65	0.44	-2.51	-0.79	14.19	< 0.001^*^
		Public	0^a^					
	No of comorbidities	≤ 1	-0.67	0.37	-1.40	0.07	3.17	0.075
		≥ 2	0^a^					
	No of dialysis sessions per month	≤ 3	0^a^					
		4–6	0.42	0.59	-0.74	1.58	0.50	0.478
		> 6	0.57	0.57	-0.55	1.69	0.99	0.319
		(Scale)	2.28^b^	0.22	1.88	2.76		

(a) Reference category; (b) Maximum likelihood estimate; *Significance at *p* < 0.05

## Discussion

This study revealed that most ESKD patients were willing to pay for haemodialysis for themselves with a mean benefit of USD 25,999.06. Previous studies have reported contrasting findings on kidney replacement therapy in Ghana [18] and chronic illnesses in Malaysia and Vietnam [33, 34]. In the current study, patients’ willingness to pay more for their use may be related to the rational expectations theory, which posits that individuals base their decisions on human rationality, information, and past experiences. The WTP is related to the individual’s assessment of the intervention’s efficacy or perceived service quality [25]. ESKD has high adverse health and non-health consequences [26]. Since haemodialysis prolongs the patient’s life and might increase the quality of life and productivity of ESKD patients [35], patients would be more likely to be willing to pay for their access to a life-saving intervention.

Nevertheless, just a quarter of ESKD patients were willing to pay the existing cost of haemodialysis and above, raising concerns about patients’ financial hardship from paying out-of-pocket. A study in Ghana similarly found that most ESKD patients would pay below the current cost of kidney replacement therapy [18]. Furthermore, over a tenth of ESKD patients were unwilling to pay for haemodialysis. Despite the high stated WTP, these findings still demonstrate financial barriers since many patients fall below the actual price tag. This highlights the need to explore public financing mechanisms rather than out-of-pocket payments to fund haemodialysis in Nigeria.

Regarding altruism, 64.8% expressed some willingness to enable access for others, with a mean value of USD 1,539.89. Our finding that many ESKD patients were willing to pay for people experiencing scarcity to access haemodialysis is consistent with evidence that social solidarity through altruistic payments could enhance equitable access to health services [36, 37]. Our finding suggests that altruism might be a feasible financing strategy to increase access to haemodialysis for low-income people. Altruistic funding is a potential strategy to improve risk- and income-cross-subsidization in national social health insurance schemes [38]. Altruistic WTP could inform the design of a sliding scale premium in social health insurance schemes that ensure wealthier people pay more for services than less wealthy people [39]. The NHIA could also promote altruistic payment for haemodialysis by leveraging the adoption model in which altruistic individuals adopt people experiencing poverty and pay their annual social health insurance premium [40]. Evaluation of altruistic WTP for the entire population is warranted to ensure evidence informs the adoption model for haemodialysis.

The current study revealed that the type of hospital ESKD patients attended determined their willingness to pay for their haemodialysis or altruistic WTP. Our finding that ESKD patients who attended private hospitals were more likely than others to be willing to pay for their haemodialysis is unsurprising because concerns about affordability in the private sector are associated with low WTP among patients with chronic illnesses [41]. The cost of haemodialysis is higher in private hospitals than in public hospitals [6]. Public hospitals tend to treat patients with lower socio-economic status and higher levels of co-morbidity than private hospitals [42]. Even when the rich and the poor seek care from private hospitals, the poor will revert to public hospitals when access inequality increase [43].

This study also showed that ESKD patients who attended public-private partnership hospitals were less likely to pay for altruistic haemodialysis. Treatment at public-private partnership (PPP) hospitals has higher out-of-pocket costs than public hospitals [6, 44], which provides contradictory incentives for people to invest additional resources in the care of others. The outsourcing fee, which represents the additional cost of providing dialysis service through the partnership, is a significant cost driver in PPP hospitals [44] and is often transferred to the patients. The government must, therefore, regulate PPP hospitals by benchmarking the outsourcing fees to ensure that service delivery through PPP hospitals results in affordable care.

This study adds to the growing literature on the WTP for kidney replacement therapy in LMICs. To our knowledge, this is the first WTP for haemodialysis in Nigeria. Overall, our findings highlight a need for policies to promote affordable access to life-saving dialysis treatment for all socio-economic groups. Nevertheless, our study has some limitations. The WTP approach relies on hypothetical scenarios and stated preferences, which may not match actual behaviour. Individuals often state higher WTP than what they would genuinely pay. This study did not verify the association between willingness to pay for haemodialysis and the actual payment. However, our study followed recommended practices, such as bidding to reach maximum WTP. Since WTP captures personal utility rather than societal value, we included altruistic WTP to account for non-use value regarding concern for others. A previous study in Nigeria showed a strong correlation between stated and actual altruistic WTP for bed nets [45]. Despite limitations, WTP remains a preferred method for valuing non-marketed benefits in health economic evaluations.

## Conclusion

This study provides new willingness-to-pay evidence regarding patient valuation of haemodialysis treatment in Nigeria, revealing that although mean WTP values were high, only a quarter of respondents were willing to pay the current costs for personal haemodialysis. Despite stated high value, the findings imply financial hardship posed by out-of-pocket expenditures, underscoring the need to develop alternative health financing mechanisms that improve affordability and equitable access through greater public subsidization and altruistic financing strategies. Options for publicly funded haemodialysis include integration into universal health coverage benefits, insurance reimbursement, and increased government budget allocations. In designing strategies to finance haemodialysis, stakeholders must pay attention to the type of hospital attended by ESKD patients, as private hospital patients had higher personal WTP while PPP hospital attendees had lower altruistic WTP. Cost-benefit analysis leveraging the willingness-to-pay data could inform tailored funding policies for haemodialysis amidst rising chronic kidney disease burden.

## Data Availability

No datasets were generated or analysed during the current study.

## Abbreviations

CKD: Chronic kidney disease
CVM: Contingent valuation method
ESKD: End–stage kidney disease
FCT: Federal Capital Territory
GDP: Gross domestic product
KRT: Kidney replacement therapy
LMICs: Low and middle–income countries
NHIA: National health insurance authority
PPP: Public–private partnership
WTP: Willingness to pay
